# Genome-wide identification analysis in wild-type *Solanum pinnatisectum* reveals some genes defending against *Phytophthora infestans*

**DOI:** 10.3389/fgene.2024.1379784

**Published:** 2024-05-15

**Authors:** Chunxiu Shen, Qineng Lu, Di Yang, Xueru Zhang, Xinping Huang, Rungen Li, Zhiqun Que, Na Chen

**Affiliations:** ^1^ Jiangxi Key Laboratory of Crop Growth and Development Regulation, College of Life Sciences, Resources and Environment Sciences, Yichun University, Yichun, China; ^2^ Grandomics Biosciences, Wuhan, China

**Keywords:** assembly, ont, resistance gene, potatoes, Hi-C technology

## Abstract

*Solanum pinnatisectum* exhibits strong resistance to late blight caused by *Phytophthora infestans* but only an incomplete genome assembly based on short Illumina reads has been published. In this study, we generated the first chromosome-level draft genome for the wild-type potato species *S. pinnatisectum* in China using Oxford Nanopore technology sequencing and Hi-C technology. The high-quality assembled genome size is 664 Mb with a scaffold N50 value of 49.17 Mb, of which 65.87% was occupied by repetitive sequences, and predominant long terminal repeats (42.51% of the entire genome). The genome of *S. pinnatisectum* was predicted to contain 34,245 genes, of which 99.34% were functionally annotated. Moreover, 303 NBS-coding disease resistance (R) genes were predicted in the *S. pinnatisectum* genome to investigate the potential mechanisms of resistance to late blight disease. The high-quality chromosome-level reference genome of *S. pinnatisectum* is expected to provide potential valuable resources for intensively and effectively investigating molecular breeding and genetic research in the future.

## Introduction

Potatoes (Solanaceae family, *Solanum* genus, *Potatoes* (G. Don) D’Arcy subgenus, *Petota Dumortier* section) originated in the Andean mountains of South America and are now cultivated across more than 160 countries and territories worldwide. As of 2018, the global potato production was an impressive 368 million tons, positioning it as the world’s fourth most significant food crop after maize, rice, and wheat (Epstein, 2014). Potatoes hold both substantial nutritional and economic value, as they not only contain starch, protein, crude fiber, and other essential nutrients but also boast carotenoids and ascorbic acid that are components not typically found in many cereal grains (Nayak et al., 2014). Potatoes can be further processed into whole flour and modified starch, which can be used as raw materials in various fields, including food industries, chemical industries, and medical treatments. China is currently the world’s largest potato producer, with the highest total annual cultivated area and overall output compared to other nations and regions (http://www.fao.org/statistics/en/). Simultaneously, advancements in potato breeding and cultivation technologies have led to a continuous rise in the yield per unit area of potatoes in China.

The late blight disease in potatoes, which is caused by the facultative parasite *Phytophthora infestans* (Nowicki et al., 2012), is a globally significant agricultural threat given its high infectivity. In the middle of the 19th century, a devastating epidemic swept through Ireland, resulting in the tragic loss of millions of lives and prompting mass emigration owing to the pervasive famine (Fry et al., 2015). Even now, potato late blight persists as one of the world’s most pernicious plant pathogens, inflicting an annual economic toll estimated at nearly 10 billion US dollars (Majeed et al., 2017). Presently, the control of potato late blight disease relies heavily upon chemical intervention. However, the inherent toxicities of these fungicides pose risks to public health and exacerbate environmental pollution even as the significant financial burden associated frequent fungicide applications constitute a pressing concern (Majeed et al., 2017). Furthermore, the imposition of stricter fungicide regulations underscores the limitations of depending solely on chemical pesticides for managing diseases such as the late blight of potato (Qin et al., 2016; Karki et al., 2020; Lal et al., 2021). The constant evolution of physiological virulence in *P. infestans* presents a persistent challenge to potato resistance breeding efforts.

It is of vital significance to develop potato resistance to late blight disease through the exploration of new resistant germplasms, research on new resistance genes, and expansion of the resource bank for resistance breeding. Resistance to the causative agent of the Irish Potato Famine, i.e., the pathogen *P. infestans*, has largely been identified within the genetic material of various wild Solanum species (Gao et al., 2020). The discovery of fresh disease-resistant genes and development of novel technologies, such as quantitative trait locus (QTL) mapping, have significantly enriched the material foundation for developing late-blight-resistant potato varieties (Danan et al., 2011; Albert et al., 2015). Petra Oberhagemann found quantitative resistance to late blight in potatoes using QTL mapping (Oberhagemann et al., 1999). A novel broad-spectrum disease resistance gene from the wild potato species *S. bulbocastanum* has been found using dRenSeq (Li et al., 2023). *S. pinnatisectum* (2n = 2x = 24), a diploid wild potato species native to Mexico, exhibits high resistance to late blight disease (Chen et al., 2003); despite this characteristic, *S. pinnatisectum* has received less attention compared to other wild relatives like *S. demissum* and *S. bulbocastanum* primarily because of the high degree of incompatibility it shares with other wild and cultivated potato types, which has historically limited its germplasm resource utilization potential.

Traditional breeding techniques still demonstrate limitations to fully exploiting available germplasm resources. Moreover, there exist varying degrees of reproductive isolation between cultivated- and wild-type potatoes largely due to disparities in the ploidy levels and endosperm balance numbers, among other factors. This inherent isolation hinders the transfer of some disease-resistance genes from wild potatoes to cultivated varieties via conventional breeding methods. However, as modern breeding technologies have overcome the reproductive isolation between the wild and cultivated varieties as well as further expanded the scope of the usable wild varieties, the disease-resistance genes of *S. pinnatisectum* can be effectively applied in disease-resistance breeding. It is indeed feasible to leverage advanced sequencing technologies at the molecular level to uncover the disease resistance traits of *S*. *pinnatisectum* and potentially other novel resistance genes. To date, only one reference genome has been published for *S. pinnatisectum* based on the short paired-end reads assembly of the next-generation sequencing (NGS) technology (Gao et al., 2020; Li et al., 2023), and the gaps in the genome sequence may subsequently hamper the discovery of potential disease-resistance genes.

Given the lengthy history of plant–pathogen interactions, plants have evolved complex defense mechanisms to perceive and counteract pathogen attacks, with a multitude of genes playing critical roles in disease resistance. Research has suggested that terpenoids may contribute significantly to this resistance in various plant species (Bell et al., 1994; Hall et al., 2011; Schmelz et al., 2014), such as rice (Yajima and Mori, 2000; Inoue et al., 2013) and cotton (Mace et al., 1976; Zhang et al., 1993; Pierce et al., 1996). Moreover, the plant cell wall and innate immunity of each cell also provide disease resistance against plant–pathogen interactions (Van Der Biezen and Jones, 1998; Dangl and Jones, 2001; Ausebel, 2005; Chisholm et al., 2006). Resistance (R) genes constitute a superfamily and famous genes are used to study disease resistance in plants (Bergelson et al., 2001; Pandolfi et al., 2017; Zhang et al., 2022) to recognize the pathogen-derived virulence factors. These R genes can directly or indirectly recognize pathogen-derived virulence factors, thereby activating a series of disease-resistance signaling pathways that ultimately lead to plant protection against diseases (Staskawicz et al., 2001; Abramovitch and Martin, 2004; Zhou and Chai, 2008). The R genes usually contain several motifs, namely, the nucleotide-binding site (NBS) and leucine-rich repeat (LRR) region, which are together referred to as the NBS-LRR genes (Bezerra-Neto et al., 2020).

## Results

### Genome estimation and assembly

**FIGURE 1:**
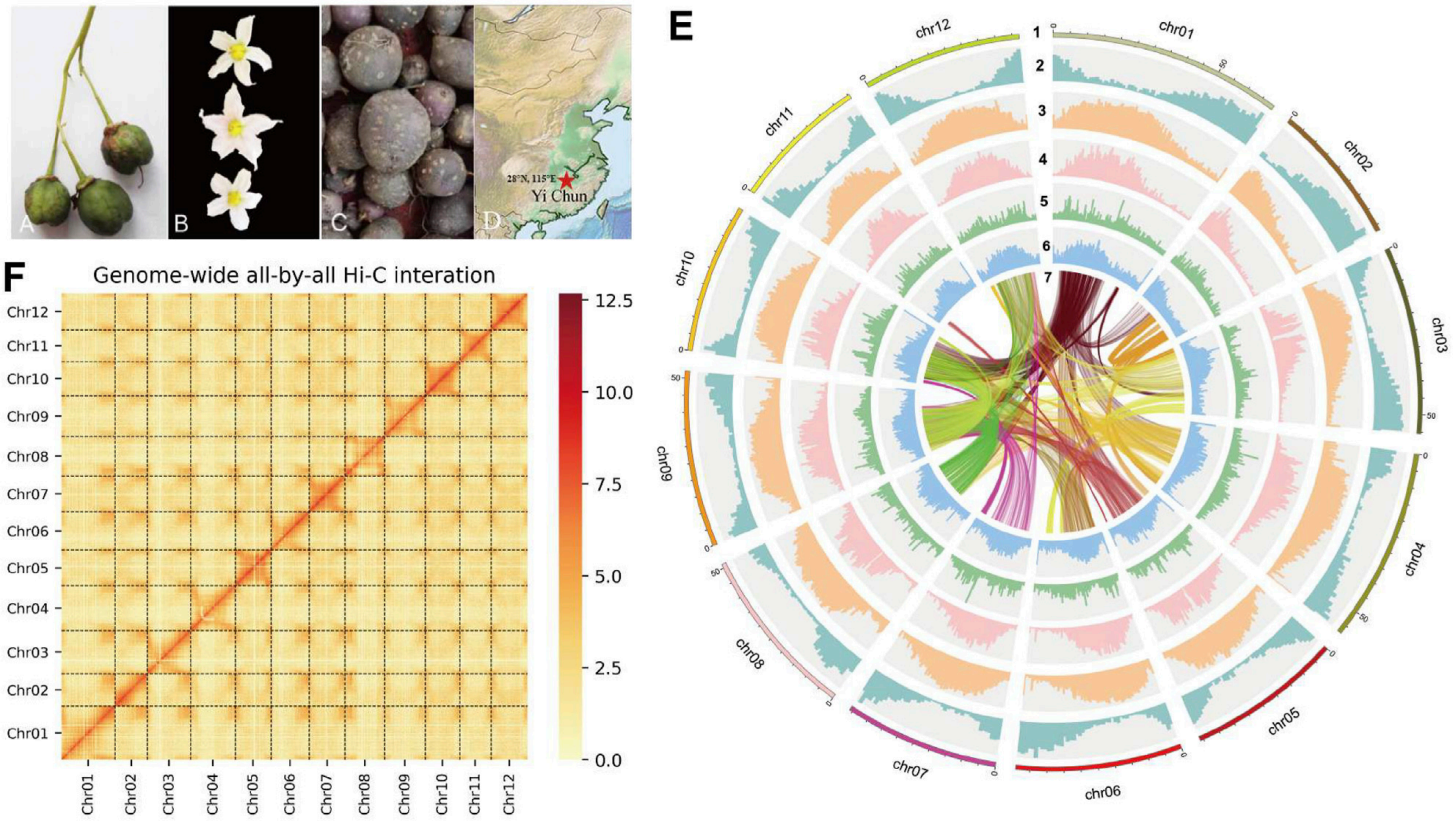
**(A)** The fruit of *S. Pinnatisectum*. **(B)** The flower of *S. Pinnatisectum*. **(C)** The tuber of *S. Pinnatisectum*. **(D)** The Red five-pointed star is the sampling site (28°N 115°E). The location is Yichun. **(E)** The *S. Pinnatisectum*’s genome information in circos. The scale of 5 Mb in the outermost circos and the chromosome was cut into 0.5 Mb to stat the density of different type among 2-6 circos. The contents represented by the circles from the outside to the inside are displayed as follows: 1: The length of chromosome; 2: The density of gene; 3: The density of total repeat sequences; 4: The density of Gypsy; 5: The density of Copia; 6: The distribution of GC base content; 7: The colinearity of gene block and the number of gene in each block is more than 40. F: The Hi-C heatmap of *S. Pinnatisectum*.

Leaves were collected from *S. pinnatisectum*, and a total of 56 Gb of the 150-bp paired-end DNA reads was obtained after adapter trimming and quality filtering (Supplementary Table: Sequence and Analysis Data). The survey analysis estimated the *S. pinnatisectum* genome size to be 664–668 Mb with a heterozygosity of 1.49% (See Supplementary Table S1; Supplementary Figure S1). Regarding the genome assembly process, given the relatively high degree of heterozygosity present within the *S. pinnatisectum* genome, we implemented a filtration step to eliminate redundant contigs and those that aligned with the mitochondrial or chloroplast sequences from the nucleotide sequence database (NT). This approach ensures a more accurate and refined representation of the genomic data. The resulting genome size was 664 Mb, with the contig N50 value being 9 Mb (Table 1). We first evaluated the quality of the assembly using benchmarking universal single-copy orthologs (BUSCO), whose results demonstrated that the assembled *S. pinnatisectum* genome exhibited a high level of completeness, as evidenced by a gene set completeness rate of 99.38% (Table 1; Supplementary Table S3). To further validate the accuracy of our assembly, we mapped the Illumina paired-end reads back to the *S. pinnatisectum* genome; the mapping rate achieved was 99.82%, and the genome coverage with a read depth of at least 5× reached 98.10% (Table 1; Supplementary Table S2). Ultimately, employing high-resolution chromosome conformation capture (Hi-C) technology allowed us to anchor the genomic contigs onto 12 chromosomes, resulting in a total length of 600.10 Mb and an impressive loading rate of 90.36% (Figure 1F; Supplementary Table S4). Notably, two of these chromosomes (chr02 and chr11) were each composed of a single contiguous contig (Supplementary Table S4). Given that both NGS and third-generation sequencing (TGS) data were used, it is expected that there should not be any homozygous variants reported from the NGS data because of the inherent heterozygosity within the *S. pinnatisectum* genome. Consequently, any detected homozygous variants were considered errors rather than genuine biological events. In conclusion, the quality assessment revealed that the genome accuracy was Q40, indicating a precision greater than 99.99% (Table 1; Supplementary Table S4), thereby underscoring the reliability and high fidelity of the assembled *S. pinnatisectum* genome.

**TABLE 1:** Statistics and quality assessments of the genome and genes.

Species name	*S*. *pinnatisectum*
Total length (bp)	664,135,451
Contig number	339
Contig N50 (bp)	9,057,158
Scaffold number	140
Scaffold N50 (bp)	49,171,345
Genome BUSCO in embryophyta_odb10 (%)	99.38
TGS mapping ratio (%)	99.63
NGS mapping ratio (%)	99.82
Error rate of homozygous variants (Coverage ≧1×) (%)	0.004025
Error rate of homozygous variants (Coverage ≧5×) (%)	0.003049
Accuracy of the genome	Q40
Number of annotated protein genes	34,245
Repeat sequence in the genome (length bp/ratio %)	437,472,471/65.87
Gene BUSCO in embryophyta_odb10 (%)	97.96

### Gene prediction and annotation

Genomic repeats were analyzed to assess the estimated genome quality that the *S. pinnatisectum* genome harbors 437.47 Mb of repetitive sequences, which account for a substantial proportion of 65.87% of the entire genomic content. Furthermore, long terminal repeats (LTRs) were found to be dominant among these genomic repeats, constituting 47.52% of the total content (Table 1; Supplementary Table S5). We proceeded to predict 34,245 protein-coding genes in *S. pinnatisectum* using a combined approach involving *ab initio*, transcriptome-based, and homology-based predictions. Of these predicted genes, an impressive 99.34% (equating to 34,019 genes) were successfully annotated with functional information across five distinct databases (refer to Table 1; Supplementary Table S6; Supplementary Figure S2). The BUSCO assessment revealed a high level of completeness of 97.96% for the protein sequences within the *S. pinnatisectum* genome (also detailed in Table 1; Supplementary Table S3). Additionally, our examination identified a comprehensive set of non-coding RNAs within the *S. pinnatisectum* genome, including 880 microRNAs, 1,036 transfer RNAs, 223 ribosomal RNAs, and 24 regulatory RNAs (the specifics can be found in Supplementary Table S7). The annotated results of the genes and repeat sequences are shown in Figure 1E. The density of repeats and genes also indicates that the genome sequence and annotated result are of high quality.

### Gene family evolution

**FIGURE 2:**
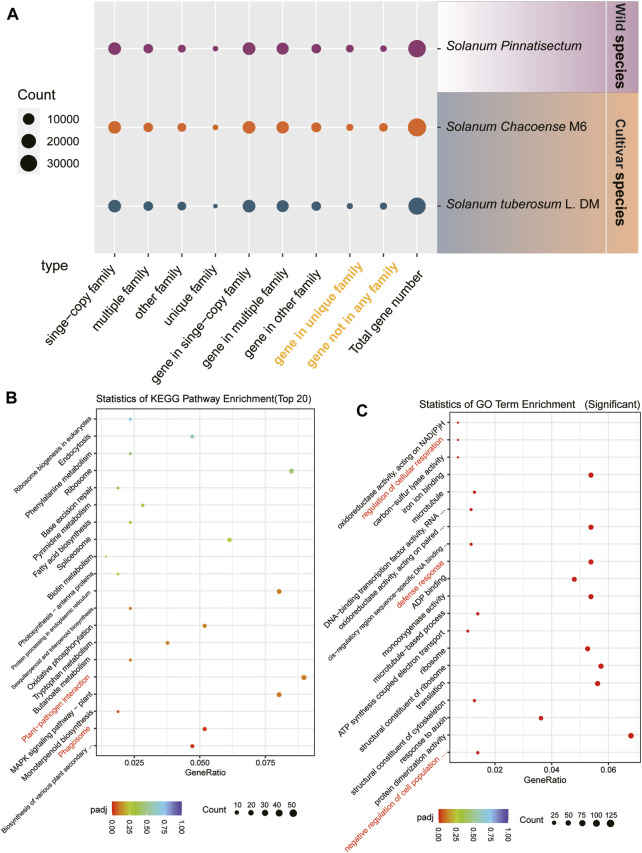
Results of the gene family. **(A)** Numbers of gene families and genes among *S. tuberosum* L. (blue), *S. chacoense* (orange), and *S. pinnatisectum* (purple). The species-specific genes are denoted in yellow words. The spot size represents the number of gene families or genes. **(B)** Top-20 significant KEGG pathways in the enrichment analysis. The spot size represents the number of each enriched pathway (see the count bar), and the color is the degree of enrichment (see p.adj bar). **(C)** Top-20 significantly enriched GO terms in the enrichment analysis. The spot and color are analyzed in the same manner as in (B).

For the gene family analysis, three potato species were considered. There are 13,765 single-copy families in the genes of *S. tuberosum* L., *S. chacoense*, and *S. pinnatisectum*. These sets of single-copy family genes separately constitute 41.90%, 41.75%, and 40.20% of the total genes. A total of 5,416 multiple-copy families were identified in each of the species, with corresponding counts of 11,466 genes in *S. tuberosum* L., 12,749 genes in *S. chacoense*, and 11,651 genes in *S. pinnatisectum*. For the remaining families, *S. tuberosum* L. accounted for 3,875, *S. chacoense* accounted for 3,888, and *S. pinnatisectum* accounted for 2,555 family groups. There were 205, 492, and 427 unique family groups specific to *S. tuberosum* L., *S. chacoense*, and *S. pinnatisectum*, respectively. Moreover, there were certain genes that were not affiliated with any recognized gene families; thus, there were 1,664 non-family genes in *S. tuberosum* L., 3,712 genes in *S. chacoense*, and 1,078 genes in *S. pinnatisectum* (as shown in Figure 2A and detailed in Supplementary Table S8).

Focusing on *S. pinnatisectum*, we selected its unique family genes (427) and non-family genes (1,078) for functional analysis, which are highlighted in yellow in Figure 2A and enumerated in Supplementary Table S8. These genes represent the species-specific repertoire compared to the two other cultivated species. To gain deeper insights into their functional significance, we conducted enrichment analyses using KEGG pathways and GO terms for these species-specific genes in *S. pinnatisectum*.

The top 20 significantly enriched KEGG pathways are presented in Figure 2B; here, aside from the essential cellular processes such as ribosomes, protein processing in the endoplasmic reticulum, and the MAPK signaling pathway specific to plants, we observed that the plant–pathogen interactions and phagosome pathways showed significant enrichment of species-specific genes in the wild-type *S. pinnatisectum*. The most significantly enriched GO terms are visually depicted in Figure 2C, where the regulation of cellular respiration, defense responses, and negative regulation of cell population functions show significant enrichment in species-specific genes in the wild type *S. pinnatisectum*. The objective of this analysis was to examine the specific roles and biological pathways potentially engaged by these distinct non-family genes within *S. pinnatisectum*.

### Differential expression gene analysis

**FIGURE 3:**
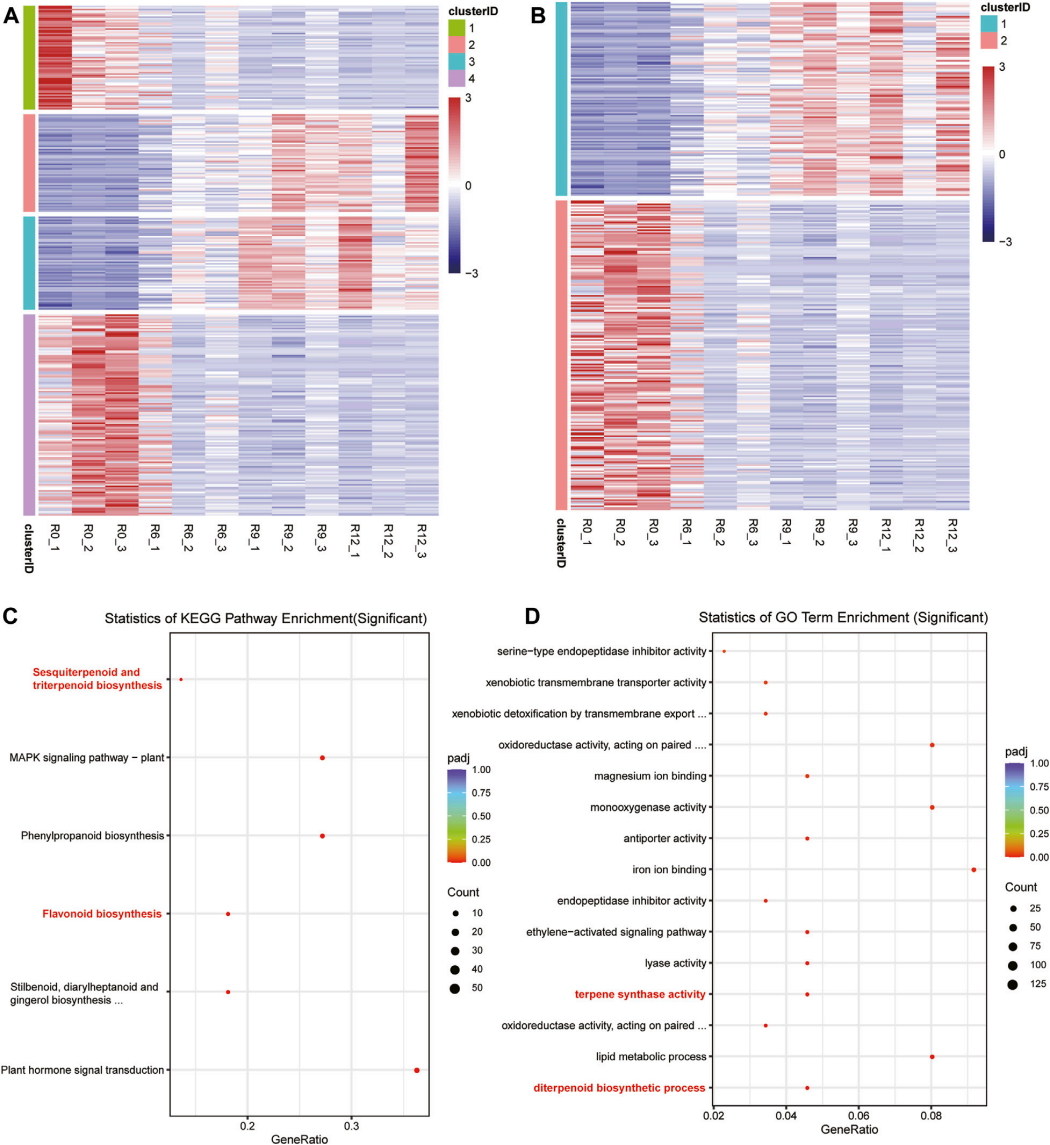
Clustering of the differential expressions and enrichment of the specific cluster genes. **(A)** Gene classification into four clusters, where blue indicates low expression and red indicates high expression. **(B)** Gene classification into two clusters. **(C)** Gene belonging to Cluster 1 in (A) with significant enrichment of the KEGG pathway in the enrichment analysis. **(D)** Gene belonging to Cluster 1 in (A) with significant enrichment of the GO terms in the enrichment analysis.

Based on the time-series materials, a time-series differential expression analysis was conducted using data collected at 0 h, 6 h, 9 h, and 12 h. This process resulted in the identification of 330 differentially expressed genes over time (refer to Supplementary Table: Cluster Analysis). Initially, these genes were categorized into four distinct groups (Figure 3A); the initial gene expressions in Clusters 1 and 4 were significantly lower than their counterparts in Clusters 2 and 3, which displayed notably heightened expressions. However, with the passage of time, an intriguing shift was observed in the expression dynamics; the transcriptional activities in Clusters 1 and 4 increased steadily, while there were concurrent reductions in the expression levels in Clusters 2 and 3. Subsequently, these four categories could be further divided into two main clusters (Figure 3B). Upon closer examination, it was observed that genes within Cluster 1 exhibited an ascending expression pattern, starting from low levels at 0 h and increasing to higher levels by 12 h. In contrast, genes in Cluster 2 demonstrated a descending trend, with initial high expression levels that gradually decreased over the same time period from 0 to 12 h. The KEGG and GO functional enrichment analyses disclosed that Cluster 1 manifested substantial overrepresentation in multiple biological pathways. Notably, these included the KEGG pathways for sesquiterpenoid, triterpenoid, flavonoid, and phenylpropanoid biosyntheses, as depicted in Figure 3C. Regarding the GO terms, Cluster 1 exhibited remarkable enrichment in terpene synthase activity, diterpenoid biosynthesis processes, and monooxygenase activities, emphasizing its unique biological roles and molecular functions. Additional details on this gene enrichment may be found in the Supplementary Table.

### Analysis of resistance-related (R) genes

**FIGURE 4:**
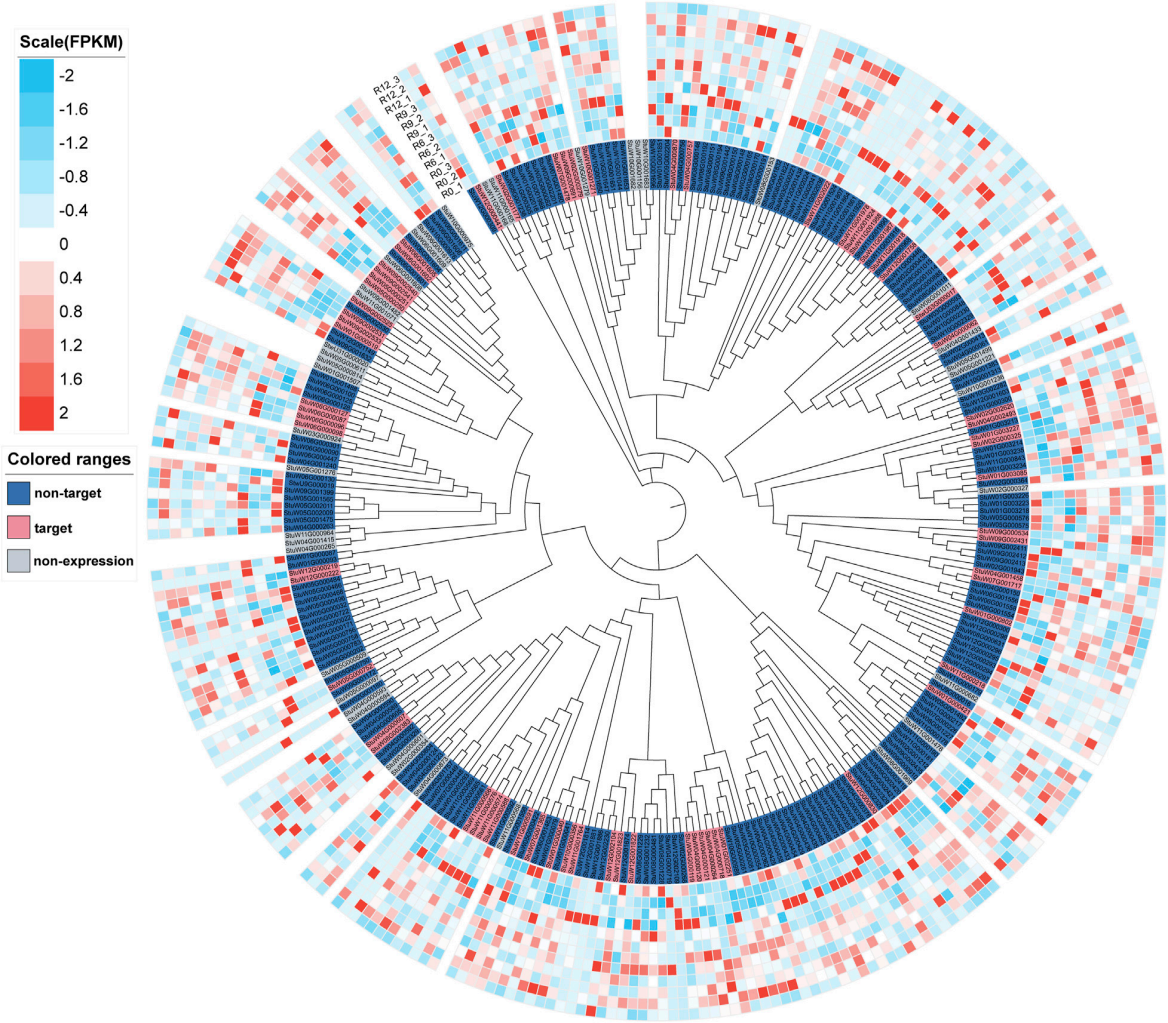
Tree and expressive heatmap of the resistance genes. The color light blue indicates low expression, and red shows high expression. The non-expression label is indicated by the gray genes, which are not expressed in 12 RNAseq. The non-target label shown in dark blue indicates the expression pattern of the gene, which is not from low to high with time lapse in the 12 RNAseq. The target label shown in pink indicates the expression pattern of the gene from low to high with time lapse in 12 RNAseq.

**FIGURE 5:**
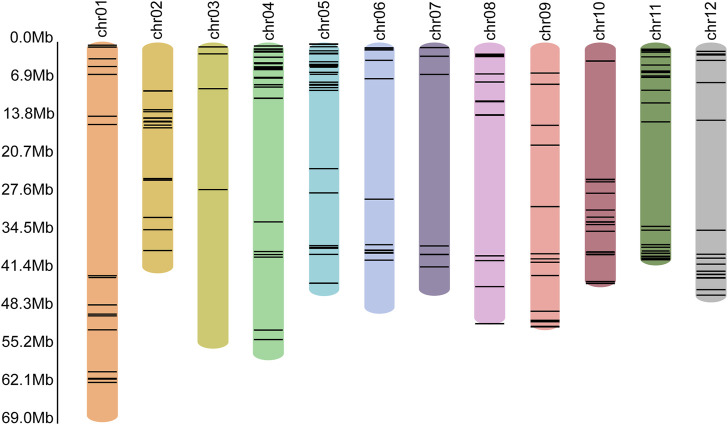
Locations of the R genes in each chromosome. The different colored bars represent different chromosomes, with the name of the chromosome shown at the top. The black lines in the colored bars show the locations of the R genes. The lengths of the colored bars represent the lengths of the corresponding chromosomes. The scale of length is shown along the left axis.

In the genome of *S. pinnatisectum,* 303 genes containing the motifs of the NB-ARC (NBS) domain were identified (Supplementary Table: R gene statistics). These protein sequences were aligned to construct the gene tree (Figure 4). The gene FPKM of the R genes based on 12 RNAseq are displayed in the outer ring of the gene tree. The differential expression ranges from −2 to 2 (see the FPKM label in Figure 4). The non-expression label (gray color) means that these genes (39 numbers) are not expressed in the 12 RNAseq (Figure 4). The target label (pink) means that these genes (68 numbers) show ascending expression patterns from low to high with time lapse in the 12 RNAseq (Figure 4). Conversely, the non-target label applies to 196 genes (indicated in dark blue), whose expression patterns do not follow the low-to-high trend in the 12 RNAseq dataset. For the motifs in the R gene, different motifs in the sequences were identified, and the number of motif genes is 10 at most (Supplementary Figure S3). These genes have different locations in the chromosome; 299 out of the 303 genes are located in the chromosome and the remaining 4 are located in the dispersed contigs (Supplementary Table: R gene statistics). Genes located on the chromosome are depicted in the chromosome diagram, and some genes such as chr4, chr5, and chr11 together constitute 16.17% (49 genes), 10.23% (31 genes), and 13.86% (42 genes) of the total number of genes (Figure 5).

To identify the shared and unique genes among the resistance genes and Cluster 1, gene annotation results were obtained (Supplementary Figure S4); the genes were exclusively assigned to a single group of 273 (constituting 90.10% of the total) in the R-resistance category, 119 (representing 93.70%) in Cluster 1, and 64 (comprising 62.75%) in Function. Additionally, there may exist genes that simultaneously belong to more than one subgroup. Our analysis showed that 30 genes bear functional significance and are related to R-resistance. Only eight genes (StuW01G003151, StuW01G003152, StuW01G003155, StuW02G002548, StuW04G001654, StuW05G001553, StuW10G002376, and StuW10G002377) were specifically found to have significant functionality within Cluster 1, as depicted graphically. It is worth noting that no genes were found to be common to both the R-resistance category and Cluster 1.

## Discussion

We report a high-quality chromosome-level reference genome for the wild-type potato *S. pinnatisectum* based on genome assessment, and the distribution of the gene and repeat sequences also prove the quality of the genome. We assembled a 664-Mb genome and annotated 34,245 protein-coding genes.

To unravel the species affinities and evolutionary processes as well as identify species-specific genes and reconstruct their evolutionary histories, the Markov clustering (MCL) algorithm has been widely and effectively employed across numerous databases (Van Dongen, 2000; Zhang et al., 2019). Previous research endeavors have successfully applied this methodology to analyze gene families across various organisms, including humans (Bac Resource Consortium et al., 2001), *Drosophila* (Clark et al., 2007), plants (Guo, 2013; Zhang et al., 2020), vertebrates, and invertebrates (Prachumwat and Li, 2008). In the present study, a considerable proportion of the major genes, approximately 40%, are categorized as single-copy families in both the wild and cultivated species. However, wild species (*S. pinnatisectum*) exhibits a significantly lower frequency of single-copy genes and harbors a larger number of unique genes. The fewer unique genes observed in the cultivated species compared to the wild counterparts may be functionally relevant, suggesting that gene loss in the cultivated species is not a random process but is rather subject to functional selection pressures (Kondrrashov et al., 2002; Blanc and Wolfe, 2004).

To reveal the possible mechanisms of resistance of *S. pinnatisectum* to late blight disease, we initially compared *S. pinnatisectum* with other cultivated species and found that the plant–pathogen interactions and phagosome pathways were significantly enriched with species-specific genes. Similarly, within the KEGG pathways, we detected analogous patterns of enrichment in both the plant–pathogen interaction and phagosome pathways. This finding points to a similar upregulation of genes linked to late blight resistance in the wild species, which corroborates previous research (Bhatia et al., 2023), underscoring the distinctive biological responses evident in *S. pinnatisectum* (Pierce et al., 1996). Meanwhile, the terms of regulation of cellular respiration, defense responses, and negative regulation of cell population were also found among the significant enrichment terms in *S. pinnatisectum* (Figure 2C) (Dangl and Jones, 2001; Abramovitch and Martin, 2004; Zhou and Chai, 2008). These pathways and terms are obviously involved in plant resistance directly or indirectly.

The special 12 RNAseq revealed that some new secondary metabolite pathways and terms, such as sesquiterpenoid and triterpenoid biosynthesis, flavonoid biosynthesis, terpene synthase activity, and diterpenoid biosynthetic. Some studies have indicated that terpenoids can protect against some diseases or are associated with resistance to pathogens in plants (Mace et al., 1976; Zhang et al., 1993; Bell et al., 1994; Yajima and Mori, 2000; Hall et al., 2011; Schmelz et al., 2014). Furthermore, R genes play an important role in resistance, so we further identified 303 resistance-related genes in *S. pinnatisectum*. Another set of 68 genes, whose the expression patterns range from low to high with time lapse in the 12 RNAseq (Figure 4), may be involved in resistance to late blight disease, as verified by the expression profiles of these genes in the transcriptome data (Van Der Biezen and Jones, 1998; Pandolfi et al., 2017; Bezerra-Neto et al., 2020; Zhang et al., 2022). The Venn diagram shows that some of the genes that enhance over time in Cluster 1 are correlated with functional annotations, with 37.3% demonstrating a connection to the R genes and Cluster 1. While there was no overlap between the resistance genes and Cluster 1, the Venn diagram demonstrated a significant relationship between the three sets of results.

The current analysis admittedly has several limitations. First, the modest sample size restricts inference since it encompasses just one wild species; broadening the scope to multiple plant species would enhance the robustness of the findings. Second, while sequencing with the Oxford Nanopore technology (ONT) offers superior comprehensiveness and longer read lengths than typical NGS, adopting HIFI data might augment the verification of our discoveries in future work. Lastly, the absence of experimental validations for the derived conclusions is a notable weakness. Integration of the root genome-wide association studies (GWS) in future investigations is expected to contribute significantly to the overall persuasiveness and impact of this research.

Summarily, the assembled genome sequence of *S. pinnatisectum* is expected to become an important complement to the genome of potato species and is expected to provide undiscovered information for further understanding of the fundamental disease-resistance mechanisms to improve molecular breeding strategies in potato plants. The genomic resource obtained herein will be potentially helpful for improving the potato quality and production in the future.

## Methods

### Genome and transcriptome sequencing

For genome sequencing, a single tissue culture seedling of *S. pinnatisectum* was collected from Jiangxi Key Laboratory of Crop Growth and Development Regulation (28^o^N 115^o^E) (Figures 1A–D). The total genomic DNA from the young fresh leaves of one plant was extracted using the CTAB method (Doyle and Doyle, 1987). Approximately 10 µg of the DNA was sheared into 10–50 kb fragments, followed by size selection on the BluePippin instrument. Approximately 5 μg of the recovered DNA was retrieved for library construction using the Ligation Sequencing 1D kit (SQK-LSK109, ONT, United Kingdom) according to manufacturer instructions, and the final library was sequenced on the Oxford Nanopore PromethION platform (ONT, United Kingdom) (Lu et al., 2016) at the Genome Center of Grandomics (Wuhan, China). Reads with mean quality scores higher than 7 were retained. For the Illumina library construction, the extracted DNA was fragmented and fractionated from the same source and was subjected to paired-end library construction, which was subsequently sequenced on the Illumina NovaSeq 6000 platform (Illumina Inc., CA, USA). Furthermore, leaves were collected from the same *S. pinnatisectum* source, and RNAseq reads were generated for genome annotation using the Illumina platform.

### Genome assembly

The Illumina paired-end reads were filtered using fastp (v0.19.6) (Chen et al., 2018) with default parameters and were then applied toward genome size and heterozygosity estimations using Jellyfish (v2.2.3) (Marcais and Kingsford, 2011). Approximately 62.59 Gb of the pass reads sequenced from the Nanopore PromethION platform by Guppy (Sherathiya et al., 2021) were obtained, and the *S. pinnatisectum* genome was subsequently assembled using NextDenovo software (https://github.com/Nextomics/NextDenovo) (read_cutoff = 1k, seed_cutoff = 28k). To obtain a genome with greater accuracy, error correction was performed on the assembled contigs using Racon (v1.5.0) (Vaser et al., 2017) with the Nanopore long reads and NextPolish (v1.2.4) (Hu et al., 2019) with the Illumina short reads for three and four rounds, respectively. The genome redundancies were detected and removed by Redundans (Pryszcz and Gabaldón, 2016) (with --identity 0.88 and --overlap 0.88).

To evaluate the accuracy of the genome assembly, the Illumina genomic paired-end reads were mapped to the genome contig sequences using the “mem” submodule of BWA (Jung and Han, 2022; Langarita et al., 2023). The mapping identity and genome coverage of the genome assembly were calculated from the mapping results obtained with SAMtools v1.4 (Li et al., 2009) with the default parameters. Homozygous single-base variations were subsequently detected using BCFtools v1.8.0 (Narasimhan et al., 2016; Danecek and Mccarthy, 2017) with the default parameters. Furthermore, the Illumina RNAseq reads were mapped to the genome sequence using HISAT2 v2.1 (Kim et al., 2019) with the default parameters, and the mapping rate of the RNAseq reads was calculated with SAMtools (Li et al., 2009). The completeness of the conserved genes and eukaryote core gene assembly were evaluated using BUSCO v5.1.3 (Simao et al., 2015; Seppey et al., 2019) with the “embryophyta_odb10” dataset.

To further eliminate contaminated sequences of the genome that could cause potential problems during downstream analysis, the error-corrected genome contigs were aligned with the Nucleotide Sequence Database (NT) (Harger et al., 2000) using BLASTN v2.9 (Camacho et al., 2009) with the parameter “E-value 1e-5,” and the sequence alignment results were classified based on species taxonomy. The contigs aligned to taxonomies except “Viridiplantae” and “Nohit” were classified as contamination sequences and filtered out from the genome assembly.

### Hi-C sequencing and chromosome construction

The Hi-C library was constructed using young fresh leaves from the same *S. pinnatisectum* Dunal and sequenced using the Illumina platform. An improved Hi-C procedure (Lieberman-Aiden et al., 2009; Louwers et al., 2009; Rao et al., 2014) was adapted. Briefly, the leaves were fixed with 1% formaldehyde to induce crosslinking (Sigma) and were subsequently lysed to form the cohesive ends by restriction endonuclease DPN II (NEB). The digested DNA was blunt-ended by filling the nucleotides by Klenow enzyme (NEB) with biotin-14-dATP (Invitrogen), followed by ligation by T4 DNA ligase (NEB). After overnight incubation to reverse the crosslinks, the ligated DNA was sheared into 300–600 bp fragments. The DNA fragments were blunt-end repaired and A-tailed, followed by purification through biotin-streptavidin-mediated pull down. Finally, the Hi-C library was sequenced on the Illumina NovaSeq-6000 platform (Illumina Inc., CA, USA). For chromosome-level scaffolding, Hi-C paired-end reads were filtered using fastp (v0.19.4) (Chen et al., 2018) with the default parameters and were then aligned to the decontaminated genome contigs using bowtie2 (v2.3.2) (Langmead and Salzberg, 2012) with the end-to-end model (-very-sensitive -L 30). LACHESIS (Burton et al., 2013) (https://github.com/shendurelab/LACHESIS) was subsequently applied according to the agglomerative hierarchical clustering algorithm to cluster the contigs with CLUSTER MIN RE SITES, CLUSTER MAX LINK DENSITY, CLUSTER NONINFORMATIVE RATIO, ORDER MIN N RES IN TRUNK, and ORDER MIN N RES IN SHREDS set to 100, 2.5, 1.4, 60, and 60, respectively, to assemble the genome contigs into groups that were further ordered and oriented into chromosomes. Finally, the chromosome-level genome was revised manually based on the heat-map matrix of Hi-C.

### Annotation of repetitive elements

Tandem repeats (TRs) across the *S. pinnatisectum* genome were annotated using GMATA (v2.2) (Wang and Wang, 2016) with the default parameters and Tandem Repeats Finder (TRF) (v4.07b) (Benson, 1999) (2 7 7 80 10 50 500 -f -d -h -r). The plant transposable elements (TEs) were searched separately using LTR_finder (v1.0.6) (Xu and Wang, 2007) and LTR_harvest (v1.6.5) (Ellinghaus et al., 2008) with the default parameters, and their results were applied to construct an LTR library file using LTR_retriever (Ou and Jiang, 2018). A MITE transposon library was generated using MITE-hunter (Han and Wessler, 2010) (-n 20 -P 0.2 -c 3) for plants and animals, and a *de novo* TE library was predicted using RepeatModeler (v1.0.11) (Bedell et al., 2000) (-engine wublast). The LTR, MITE transposon, and *de novo* TE libraries as well as Repbase database (Jurka et al., 2005) were combined to construct the final TE library for *S. pinnatisectum*, which was then used as the repeat library for RepeatMasker (Bedell et al., 2000) (v4.0.6; www.repeatmasker.org) (nolow -no_is -gff -norna -engine abblast -lib lib) to identify the TE elements in the *S. pinnatisectum* genome. The results of the TRs and TEs were merged and masked from the genome sequence. Finally, further repetitive sequences in the masked genome were found using RepeatProteinMask.

### Gene prediction

Gene models of the *S. pinnatisectum* genome were constructed by *ab initio*, homology-based, and transcriptome-based predictions. The RNAseq paired-end reads were mapped to the *S. pinnatisectum* genome using HISAT2 (v2.1.0) (Kim et al., 2019), and StringTie (v1.3.3) (Pertea et al., 2015; Pertea et al., 2016) with the default parameters was subsequently applied to assemble the transcripts that were then used as the inputs to PASA (v2.0.2) (Haas et al., 2003) (--ALIGNERS gmap, blat) for transcriptome-based gene prediction. Augustus (v3.3.1) (Stanke et al., 2006) was used for the *ab initio* gene prediction with default parameters. Moreover, the protein sequences of six homologous species (*Nicotiana attenuata, Arabidopsis thaliana, S. aethiopicum, S. pennellii, Caosicum baccatum*, and *S. chacoense*) were used for the homology-based prediction through GeMoMa (v1.5.3) (Keilwagen et al., 2019) with default parameters. The gene prediction results from the three methods were integrated using EVidenceModeler (EVM; v1.1.1) (Haas et al., 2008) to obtain the raw gene set. Finally, the genes whose sequences were composed of TEs were filtered using TransposonPSI (Urasaki et al., 2017) (http://transposonpsi.sourceforge.net). The completeness of the predicted genes was evaluated using BUSCO v5.1.3 (Simao et al., 2015; Seppey et al., 2019) with the “embryophyta_odb10” dataset.

### Non-coding RNA prediction

The annotation of the non-coding RNA set was performed next, and the genome of *S. pinnatisectum* was aligned to the non-coding database Rfam (v11.0) (Griffiths-Jones et al., 2005) using INFERNAL (Nawrocki et al., 2009) to annotate the genes of the small nuclear RNAs (snRNAs) and microRNAs (miRNAs). Transfer RNAs (tRNAs) were predicted using tRNAscan-SE (v1.3.1) (Griffiths-Jones et al., 2005). Finally, the ribosome RNAs (rRNAs) were predicted using RNAmmer (v1.2) (Lagesen et al., 2007).

### Gene functional annotation

The biological functions of the predicted genes in the *S. pinnatisectum* genome were annotated using two strategies with protein sequences. First, the predicted protein sequences were aligned with the Swiss-Prot protein database (Bairoch and Apweiler, 2000; Boutet et al., 2007), non-redundant protein sequence database (NR) (Harger et al., 2000), Kyoto Encyclopedia of Gene and Genomes (KEGG) database (Kanehisa and Goto, 2000; Kanehisa et al., 2017), and Eukaryotic Orthologous Groups (KOG) of protein database (Tatusov et al., 2003) using BLASTP (v2.7.1) (Camacho et al., 2009) with parameters “-evalue 1e-5, -max_target_seqs 1.” The Gene Ontology (GO) (Ashburner et al., 2000) analysis was subsequently performed using InterProScan (Zdobnov and Apweiler, 2001) v5.32-71.0 with the default parameters and databases. For the circles, all proteins were aligned against each other using BLASTP (-e 0.01), and Python package MCScanX (Wang et al., 2012) was used to find the collinear segments based on the protein alignment files. Subsequently, the number of genes in the collinear block was found to be more than 40, which was retained in Figure 1E.

### Transcriptome data analysis

The RNAseq data of *S. pinnatisectum* were obtained from NCBI with the BioProject accession number PRJNA616420 (*S. pinnatisectum* sample accession numbers: SRX8168235, SRX8168240, SRX8168241, SRX8168242, SRX8168243, SRX8168244, SRX8168245, SRX8168246, SRX8168247, SRX8168248, SRX8168249, and SRX8168250) (Gu et al., 2020). These data were based on histological observations of infected leaf tissues 0, 6, 9, and 12 hours post inoculation (hpi) as the time points to investigate the transcriptional dynamics of *S. pinnatisectum*. Quality control of the RNAseq reads were performed using fastp (Chen et al., 2018) with the default parameters and mapped to the *S. pinnatisectum* genome sequence using HISAT2 (v2.1.0) (Pertea et al., 2016; Kim et al., 2019) with the default parameters. Read alignments for the transcripts in each sample were extracted and counted using StringTie (v1.3.3) (Pertea et al., 2015; Pertea et al., 2016). The expression level of each gene was measured in terms of the fragments per kilobase per million (FPKM) values estimated in StringTie. The read count of each gene generated by StringTie script was used for differential expression analysis. DESeq2 (Love et al., 2014) was employed in this analysis with false discovery rate (FDR) ≤0.05 and fold change ≥2. Because the sample of RNAseq is time ordered, the genes with significantly different expressions in the time series were selected to determine the gene clusters using pheatmap with kmeans. Two and four clusters (k = 2 or k = 4) were implemented, and two clusters were found to be more suitable by comparing the two results. Thus, the gene set with low to high expressions from 0 to 12 hpi was selected for enrichment analysis through GO and KEGG using the R-package of clusterProfiler (Yu et al., 2012; Wu et al., 2021). Finally, ggplot2 (Wickham, 2009) was used to draw the bubble diagram.

### Gene family analysis

Diploid cultivated potatoes *S. tuberosum* L. (http://spuddb.uga.edu/dm_v6_1_download.shtml) and *S. chacoense* (http://spuddb.uga.edu/M6_v5_0_download.shtml) were downloaded for the downstream analyses. In these downloaded and *S*. *pinnatisectum* data, the longest mRNA in each gene was selected from the annotated file and translated to a protein sequence. Then, all selected protein sequences were aligned and clustered using Orthofinder v2.5.4 (Emms and Kelly, 2019) (-S diamond). Next, the clustered gene family was classified into four groups. The single-copy family showed that there was only one gene from each species in this group. Multiple families would indicate that the number of genes, which could be from any one species, is equal or greater than one and is not same as the single-copy family in this group. A unique family indicates that the number of genes is greater than one for any single species and that the others are zero. The family that does not belong to the single-copy, multiple, or unique categories is called as the other family. Meanwhile, there may still be some genes that may not belong to the above gene families. According to the above classification method, all genes were categorized into one of these five groups as four gene families and one non-family. For each species, the genes of the unique family and non-family are the species-specific genes. Thus, the species-specific genes in *S. pinnatisectum* were collected for gene set enrichment analysis, including GO and KEGG, using the R-package of clusterProfiler. Finally, ggplot2 was used to draw the bubble diagram.

### Resistance-related (R) gene identification

The hidden Markov model (HMM) file of the NB-ARC (NBS) domain (PF00931: http://pfam-legacy.xfam.org/search/keyword?query=PF00931) (Pandolfi et al., 2017) was download from the pfam database (http://pfam-legacy.xfam.org/) (Mistry et al., 2021). Then, the predicted protein sequences from the *S. pinnatisectum* genome were first aligned against the HMM of PF00931 using hmmscan in HMMER v3.3.2 (Potter et al., 2018). Next, from the raw aligned results, a high-quality protein set (E-value < 1×e^−20^) was selected to construct the species-specific HMM file using hmmbuild in HMMER v3.3.2 (Potter et al., 2018). Then, the predicted protein sequences from the *S. pinnatisectum* genome were aligned again with the species-specific NBS HMM using hmmscan in HMMER v3.3.2 (Potter et al., 2018). The genes with E-values less than 0.01 were obtained for the newly aligned file as the final resistance-related (R) genes (Lozano et al., 2015).

For NBS encoding proteins, the sequences were aligned using mafft v7.471 (Katoh et al., 2002) with default parameters, and the alignment file was subsequently input into Gblock v0.19b (Talavera and Castresana, 2007) (-t = p -b5 = h) to product the trimmed alignment file. Then, iqtree v2.2.0 (Nguyen et al., 2015) (-b 1000) was used to construct the gene tree with the trimmed alignment file. Based on the transcriptome data analysis, the FPKM of the R genes was obtained and then modified in the tree using iTOL (https://itol.embl.de/) (Letunic and Bork, 2007; Letunic and Bork, 2016).

## Data Availability

The datasets presented in this study can be found in online repositories. The name(s) of the repository/repositories and accession number(s) can be found in the article/Supplementary Material.

## Supplementary material

SUPPLEMENTARY FIGURE S1 Distribution of the 21-mer from NGS data. The heterozygous peak has a coverage of 35.5 and heterozygosity of 1.49%. The main peak has a coverage of 71.

SUPPLEMENTARY FIGURE S2 Gene annotated results from different databases.

SUPPLEMENTARY FIGURE S3 Motif of the R gene. Different color bars represent the different motifs, and the most motifs in a single gene is 10. The length of the line in the tree label indicates the protein sequence length.

SUPPLEMENTARY FIGURE S4 Genes in the R gene, Cluster 1, and gene annotation.

SUPPLEMENTARY TABLE S1 Genomes estimated using genomescope2 with the 21-mer.

SUPPLEMENTARY TABLE S2 Detailed results of the genome evaluation.

SUPPLEMENTARY TABLE S3 BUSCO results of the genome sequences and gene set.

SUPPLEMENTARY TABLE S4 Statistics of the Hi-C assembly.

SUPPLEMENTARY TABLE S5 Statistics of the repeat sequence annotated results.

SUPPLEMENTARY TABLE S6 Assessment of the gene annotated results.

SUPPLEMENTARY TABLE S7 Statistics of the non-coding RNA.

SUPPLEMENTARY TABLE S8 Results of gene family analysis.
