# Supplementary figures and images for: Genome-wide identification analysis in wild-type Solanum pinnatisectum reveals some genes defending against Phytophthora infestans
Source: Front Genet. 2024 May 15;15:1379784. doi: 10.3389/fgene.2024.1379784 (PMC11134371; doi:10.3389/fgene.2024.1379784)

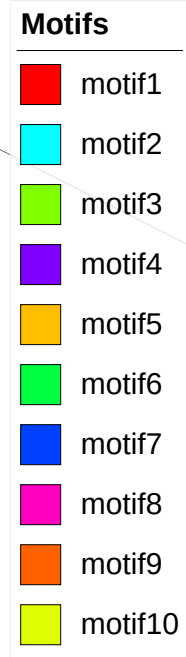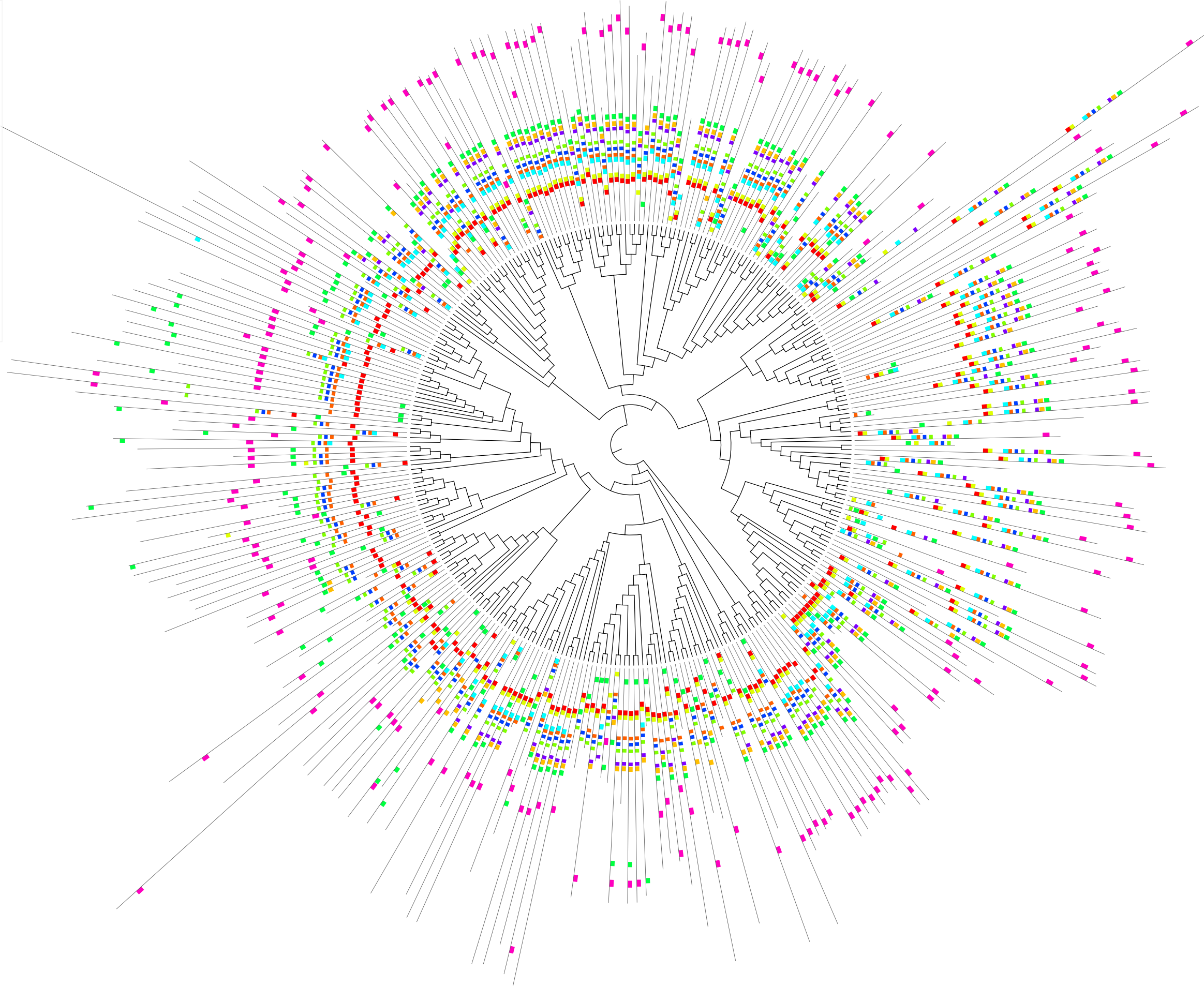

Supplement: Supplementary file 5 [file Image4.PDF]

# GenomeScope Profile

len:686,132,584bp uniq:53%

aa:98.5% ab:1.49%

kcov:35.5 err:0.19% dup:0.785 k:21 p:2

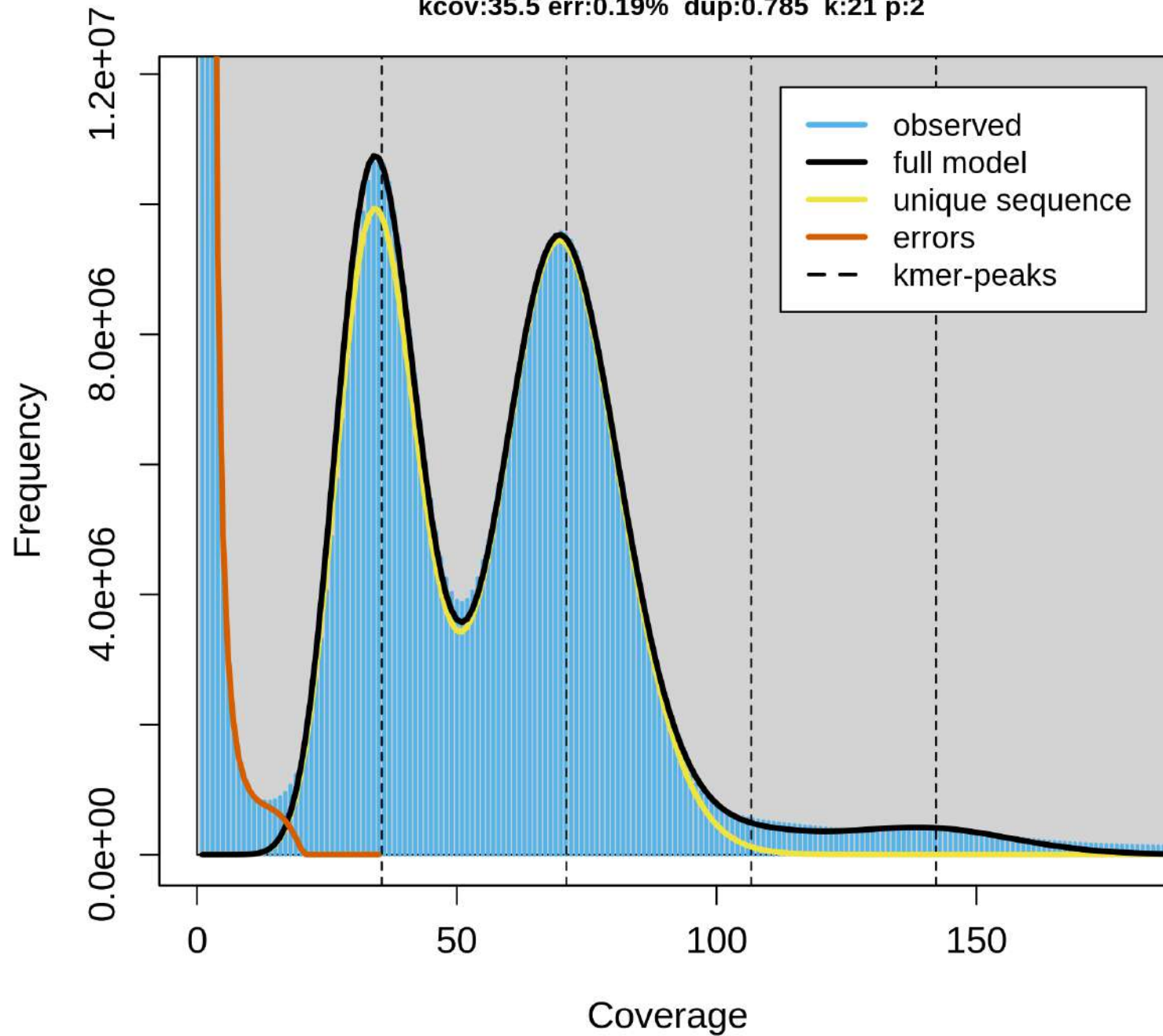

Supplement: Supplementary file 6 [file Image2.PDF]

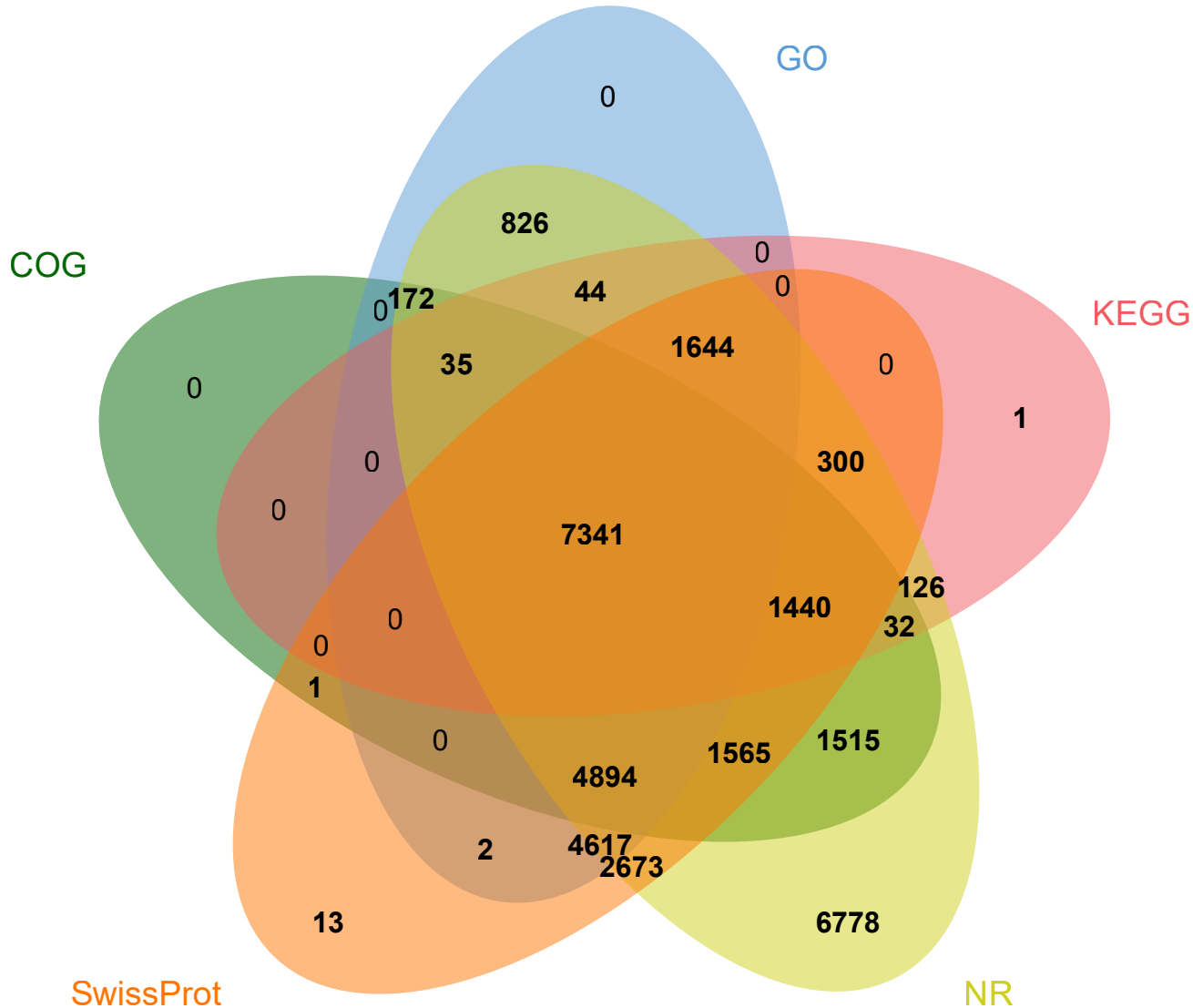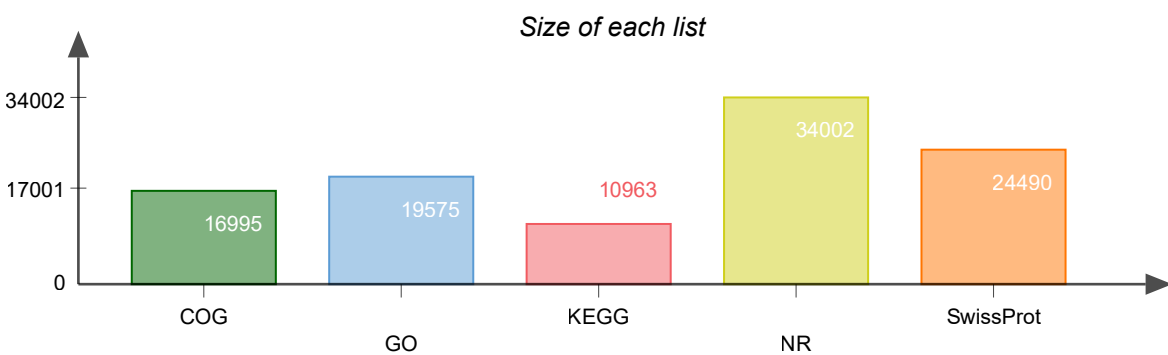

*Number of elements: specific (1) or shared by 2, 3, ... lists*

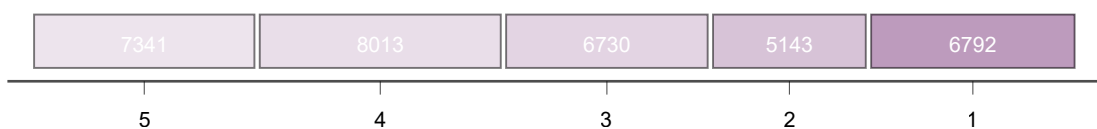

Supplement: Supplementary file 7 [file Image3.PDF]

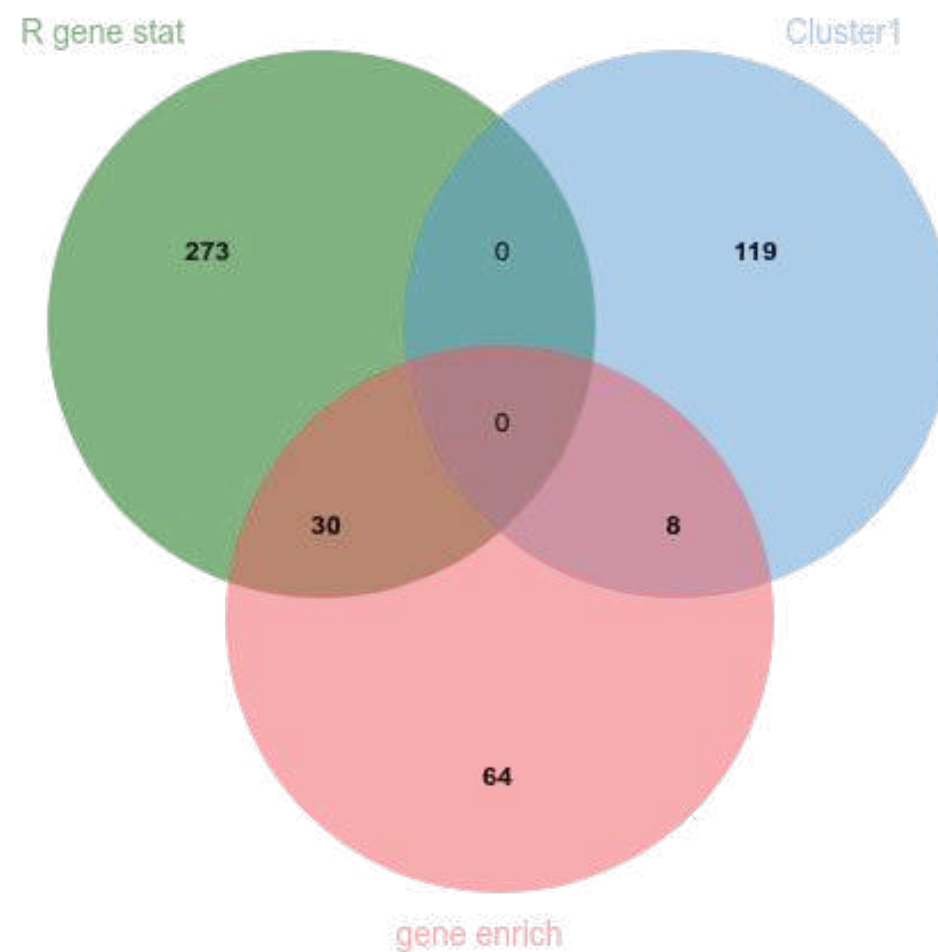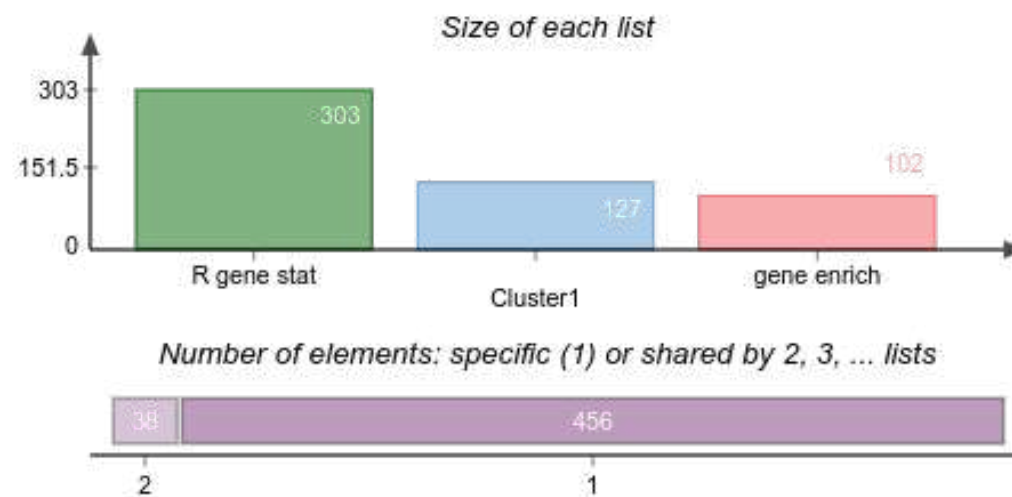

Supplement: Supplementary file 8 [file Image1.PDF]
